# General Medicine and Therapeutics

**Published:** 1893-07

**Authors:** 


### GENERAL MEDICINE AND THERAPEUTICS.

EDITED BY DR. LUTHER B. GRANDY.

### LAWSON TAIT ON THE TREATMENT OF PERITONITIS.

Years ago I was driven to the determination to discontinue the use of opium by the mouth after abdominal operations, for two reasons; first, its action on the intestines is most certainly to modify and even suspend vermicular movements; and secondly, it masks the real condition of the patient. One dose of morphine, given under the skin immediately after the operation, is all that my patients ever get, and the bulk of them do not get that.

Regarding the intolerable thirst that follows the opening of the peritoneal cavity, I have come to look upon it not as an indication for the administration of fluids, but rather for withholding them Ice is one of the things that should be banished absolutely from the sick room. It never acts in any other way than to increase thirst.

Again, if nausea sets in on the third or fourth day, or at any time after, all food (and in that I include water) is absolutely stopped for twelve hours or even longer if necessary. I was driven to this by the uniform experience that all drugs employed for the arrest of vomiting were absolutely futile; that any food given came back only altered by biliary admixture. Further, I was influenced by the perfect certainty that nothing could possibly be digested or absorbed by the stomach so long as bile was being poured into it.

I have therefore a belief that the starvation and withholding of fluid prevents the mechanical stasis of the circulation in the intestinal coats, which appears to me to be the initial stage of the fatal process of peritonitis, and this preventive measure I endeavor to assist by stimulating the peristaltic movement. I have tried a vast number of different kinds of enemata—some suggested by own thought and others suggested by ingenious friends—but I have always gone back to soap and turpentine. It is the business of any nurse watching one of my abdominal sections to note every six hours a set of four conditions—the pulse, the temperature, the occurrence of distention, and the passage of the flatus *per anum.* So soon as the latter is freely and naturally established our anxiety ends, though our watchfulness is unremitting. The want of such passage for twenty-four hours after an operation, especially if accompanied by the slightest suspicion of distention, is dealt with without fail by the nurse herself, on her own responsibility, by the administration of a turpentine enema. If the turpentine does not answer the nurse reports, and a mild saline purgative is ordered—generally a seidlitz powder—and this is repeated every four hours until it acts. If the distention increases we never rest until we have had the bowels moved, and then our anxiety is nearly always at an end and our efforts rewarded by recovery. But above all things there must be no time lost, and nobody who may be called into consultation by anxious friends must be permitted to write a prescription and try some favorite mixture.

When called into a case of well-established peritonitis I always urge a trial of this treatment, because the stage may not have passed at which it may still be effective, but the chances are that it has. I have never said that the purgative treatment will cure peritonitis, for peritonitis, once it is completely established, is a practically incurable disease, and almost uniformly fatal. Of this I am certain, if you subject the patients (not the peritonitis) to the purgative treatment, the number who will go on to incurable peritonitis will be fractional compared to what will be the result if they are left alone or submitted to any other treatment.

The practical outcome of my empirical experience is this, that the purgative treatment of peritonitis or threatening peritonitis, if it be promptly brought to bear on the case, will gain the all-important time, which will eventually turn the scale in the favor of the patient.—*British Medical Journal.*

---

### Examination of Urine for Life Insurance.

*Rules:* 1. If albumin is found in the urine, do not recommend the applicant for insurance because the quantity of albumin present is small, even though it be mere traces.

2. If albumin is present in the urine and the applicant is over forty years of age, decline the application.

3. If albumin and renal casts are found in the urine, decline the application regardless of the age of the applicant or the quantity of albumin present.

4. If albumin is found in the urine in large amounts—two or more grammes to the litre—decline the application.

5. If the applicant is of middle age or over and has always been a generous eater, especially of meat; and if he rises regularly at night to void considerable quantities of clear urine of low specific gravity; and if, in addition, there is decided tension of his pulse, with accentuation of the second sound of the heart, decline the application, even though the urine is free from albumin.

6. If true renal casts are unmistakably present in the urine, either epithelial, granular, fatty, hyaline, or composite, decline the application, even though the urine is free from albumin.

7. If the specific gravity of the urine is normal (1.020) or above, but it contains albumin at times, while at other times it contains none, especially on rising in the morning, and no casts are present in the urine of an applicant who is under thirty years of age and apparently in good health, the albuminuria is doubtless of the so-called functional form, and, in the discretion of the home office, the application may be accepted for a ten-years' endowment policy. As yet, however, such risks cannot be considered altogether safe for life policies.

8. If the applicant is subject to frequent or occasional attacks of gravel—one or more of which was recent—the application should be declined.

9. If the applicant has had one or more attacks of gravel, and more or less dull pain is present in the renal region, and the urine is more or less turbid from the presence of pus, the application should be declined.

10. If the applicant has had attacks of gravel, but five years have elapsed since the last attack, the urine remaining perfectly normal, and no pain is present in the region of the kidney, the application may be accepted.

11. If the applicant is over fifty years of age and voids his urine with more or less slowness and difficulty at times, the stream being small, forked, or dropping, and at times involuntarily shutting off before the finish, and if he rises regularly at night to void urine and is subject to periodical attacks of frequent urination, the application should be declined, even though the urine itself is in every respect normal.

12. If the urine contains sugar, the application should be declined.

13. If the urine is turbid from admixture with pus or blood, the application should be declined.—*Dr. Chas. W. Purdy.*

---

### SOME OF THE THERAPEUTIC WORK OF THE PAST YEAR.

Arsenite of copper in anæmia, the use of atropine as a hæmostatic, and the value of camphorated oil in cases of collapse have received attention. The administration of oxygen in various acute respiratory affections led to numerous communications; it was employed together with strychnine in pneumonia, alone in a severe case of broncho-pneumonia following influenza, and it was also recommended in asthma and in convalescence—massage, electricity, and oxygen being regarded as substitutes for change, exercises and sea-air. Rectal antiseptic injections in epidemic influenza, and in advanced phthisis with large cavities, have once more received commendation. Phthisis has also been treated with creosote, guaiacol, camphoric acid, and cantharidinates, but increased experience with the last named has given rise to some anxiety, owing to the frequency of consecutive albuminuria. In the treatment of vomiting, hydrochloric acid and strontium bromide have been recommended; chlorobrom has been used for seasickness and solanine for painful disorders of the stomach; orexin hydrochlorate has somewhat gained in favor as a stomachic and aid to digestion; salicylate of bismuth has been used in infantile diarrhœa, and lactic acid in many other forms of diarrhœa, having given good results even in phthisis. Much has been written of the value of glycerine in the treatment of hepatic colic, for which, when due to gall-stones, large doses of olive oil have also been recommended. Thymol has been vaunted as an anthelmintic, but its range of application appears to be very restricted.—*Medical Record.*

